# Role of 17*β*-Estradiol on Cell Proliferation and Mitochondrial Fitness in Glioblastoma Cells

**DOI:** 10.1155/2020/2314693

**Published:** 2020-02-14

**Authors:** Carlo Castruccio Castracani, Lucia Longhitano, Alfio Distefano, Daniela Anfuso, Stavroula Kalampoka, Enrico La Spina, Marinella Astuto, Roberto Avola, Massimo Caruso, Daria Nicolosi, Cesarina Giallongo, Daniele Tibullo, Giovanni Li Volti

**Affiliations:** ^1^Department of Biomedical and Biotechnological Sciences, University of Catania, Via S. Sofia 97, 95125 Catania, Italy; ^2^Department of Surgery “G. Ingrassia”, University of Catania, Via S. Sofia 87, 95125 Catania, Italy; ^3^Department of Surgery and Surgical Specialties, University of Catania, Via S. Sofia 87, 95125 Catania, Italy

## Abstract

Gliomas are the most common primary tumors of the central nervous system (CNS) in the adult. Previous data showed that estrogen affects cancer cells, but its effect is cell-type-dependent and controversial. The present study aimed to analyze the effects of estradiol (E2, 5 nM) in human glioblastoma multiforme U87-MG cells and how it may impact on cell proliferation and mitochondrial fitness. We monitored cell proliferation by xCELLigence technology and mitochondrial fitness by assessing the expression of genes involved in mitochondrial biogenesis (PGC1α, SIRT1, and TFAM), oxidative phosphorylation (ND4, Cytb, COX-II, COX IV, NDUFA6, and ATP synthase), and dynamics (OPA1, MNF2, MNF1, and FIS1). Finally, we evaluated Nrf2 nuclear translocation by immunocytochemical analysis. Our results showed that E2 resulted in a significant increase in cell proliferation, with a significant increase in the expression of genes involved in various mechanisms of mitochondrial fitness. Finally, E2 treatment resulted in a significant increase of Nrf2 nuclear translocation with a significant increase in the expression of one of its target genes (i.e., heme oxygenase-1). Our results suggest that E2 promotes proliferation in glioblastoma cells and regulate the expression of genes involved in mitochondrial fitness and chemoresistance pathway.

## 1. Introduction

Gliomas are the most common primary tumors of the central nervous system (CNS) in the adult. Glioblastoma is the most frequent and aggressive brain tumor in humans with a median survival from 14 to 17 months after the diagnosis [1, 2]. Targeted therapies directed to ubiquitous cancer-associated targets (i.e., erlotinib and gefitinib) had limited success [3–5], further reinforcing the need for the identification of glioma-specific novel molecular targets. With the advent of new technologies, several recent studies have reiterated the importance of metabolic reprogramming in various cancers. The importance of glycolysis in the survival and progression of certain cancers is undeniable, and it is increasingly evident that cancer cells may use many alternative metabolic pathways to drive their phenotype [6]. Previous data showed that estrogen affects glioblastoma cells since certain glioblastomas express estrogen receptors (ERs) [7, 8]. Consistently with this evidence, the ER-modulator tamoxifen inhibits the growth of certain glioblastomas [9–11].

Furthermore, a previous study showed that high concentrations of 17-*β*-estradiol causes apoptosis in the human breast cancer cell line MCF-7: this result is not shown with low growth-stimulated conditions in the ER-negative human breast cancer cell line MDA-MB 231 [12]. In addition to its nuclear functions, estradiol also plays an essential role in the mitochondria. The mitochondrial electron transport chain comprises several complexes formed by proteins that are encoded by the nuclear or mitochondrial genome. Moreover, E2 plays a role in mitochondrial bioenergetic function, modulating the microviscosity of the inner membrane [13] and inducing mitochondrial biogenesis genes in hepatic cells [14]. Others reported that long-term E2 treatment increased nuclear respiratory factor-1 (NRF-1) protein in cerebral blood vessels of ovariectomized rats [15]. Interestingly, high estradiol concentrations (about 10-8 M) decrease the mitochondrial DNA contents, and ATP formation and these effects were not showed at minor concentrations [16, 17]. For this reason, in this work, we study the effect of estradiol at low concentrations.

Given that, estradiol may produce cell growth or death under different conditions, depending on the expression of ERs in the brain and other tissues and the concentration of estradiol [18–20]. We analyzed the effects of estradiol in human glioblastoma multiforme U87-MG cells and how it may impact on cell proliferation and mitochondrial fitness.

## 2. Materials and Methods

### 2.1. Cell Culture and Pharmacological Treatments

Human glioblastoma cells (U87-MG) were purchased from ATCC Company (Milan, Italy). Cells were suspended in DMEM (Gibco, Cat. # 11965092) culture medium containing 10% fetal bovine serum (FBS, Gibco, category no. 10082147), 100 U/mL penicillin, and 100 U/mL streptomycin (Gibco, category no. 15070063). At 80% confluency, cells were passed using trypsin-EDTA solution (0.05% trypsin and 0.02% EDTA, Gibco, category no. 25300054) [21]. 20 *μ*g/mL 17*β*-estradiol (E2) (category no. E2758 Sigma-Aldrich, Milan, Italy) solution was prepared in 1 mL absolute ethanol (category no. 51976 Sigma-Aldrich, Milan, Italy), and it was added separately to the cell culture of all experiments at final concentrations of 5.0 nM.

### 2.2. Real-Time Monitoring of Cell Proliferation

xCELLigence experiments were performed using the RTCA (Real-Time Cell Analyzer) DP (Dual Plate) instrument according to manufacturers' instructions (Roche Applied Science, Mannheim, Germany, and ACEA Biosciences, San Diego, CA). The RTCA DP instrument includes three main components: (i) RTCA DP analyzer, which stays inside a humidified incubator maintained at 37°C and 5% CO_2_, (ii) RTCA control unit with RTCA software preinstalled, and (iii) E-plate 16 for proliferation assay. First, we defined the optimal seeding number by cell titration and growth experiments to obtain a significant cell index value and a constant cell growth (data not shown). We added 100 *μ*l of cell culture media in the E-plate 16, and we left it in the tissue culture hood for 30 minutes at room temperature: this procedure ensures the equilibrium between the culture media and E-plate surface. We inserted the E-plate 16 into a cradle pocket of the RTCA DP analyzer, and we performed blank reading to measure the background impedance of cell culture media. We added 100 *μ*l of a cell solution with a final concentration of 2500 cells/well in the E-plate 16, and, as recommended, we waited 30 minutes before starting the automatic monitoring every 15 min for 24 h.

### 2.3. Real-Time PCR for Gene Expression Analysis

RNA was extracted by Trizol® reagent (category no. 15596026, Invitrogen, Carlsbad, CA, USA). The first-strand cDNA was then synthesized with High-Capacity cDNA Reverse Transcription kit (category no. 4368814, Applied Biosystems, Foster City, CA, USA). High cDNA quality was checked, taking into consideration the housekeeping gene Ct values. Quantitative real-time PCR was performed in Step-One Fast Real-Time PCR system, Applied Biosystems, using the SYBR Green PCR MasterMix (category no. 4309155, Life Technologies, Monza, Italy). The specific PCR products were detected by the fluorescence of SYBR Green, the double-stranded DNA binding dye. Primers were designed using BLAST® (Basic Local Alignment Search Tool, NBCI, NIH), considering the shortest amplicon proposed: primers' sequences are shown in Table 1, and *β*-actin was used as the housekeeping gene. Primers were purchased by Metabion International AG (Planneg, Germany). The relative mRNA expression level was calculated by the threshold cycle (Ct) value of each PCR product and normalized with *β*-actin by using a comparative 2^‐ΔΔ*C*t^ method.

### 2.4. Immunocytochemistry

Cells were grown directly on coverslips before immunofluorescence and treated with 17*β*-estradiol (E2) at the final concentration of 5 nM. After washing with PBS, cells were fixed in 4% paraformaldehyde (category no. 1004968350 Sigma-Aldrich, Milan, Italy) for 20 min at room temperature. Subsequently, cells were incubated with primary antibody against TFAM at dilution 1 : 200, overnight at 4°C. The next day, cells were washed three times in PBS for 5 min and incubated with secondary antibodies: TRITC (anti-goat, Santa Cruz Biotechnology, Santa Cruz, CA, USA) at dilution 1 : 200 for 1 h at room temperature. The slides were mounted with medium containing DAPI (4′,6-diamidino-2phenylindole, category no. sc-3598, Santa Cruz Biotechnology, Santa Cruz, CA, USA) to visualize nuclei. The fluorescent images were obtained using a Zeiss Axio Imager Z1 microscope with Apotome 2 system (Zeiss, Milan, Italy). As a control, the specificity of immunostaining was verified by omitting incubation with the primary or secondary antibody. Immunoreactivity was evaluated considering the signal-to-noise ratio of immunofluorescence.

### 2.5. Statistical Analysis

Statistical analysis was performed using SPSS11.0 software. Statistical significance (*p* < 0.05) of differences between experimental groups was determined by the Fisher method for analysis of multiple comparisons. For comparison between treatment groups, the null hypothesis was tested by either single-factor analysis of variance (ANOVA) for multiple groups or the unpaired *t*-test for two groups, and the data are presented as mean ± SD.

## 3. Results

### 3.1. E2 Induces Glioblastoma Cell Proliferation and Mitochondrial Metabolism Gene Expression

**Figure 1:**
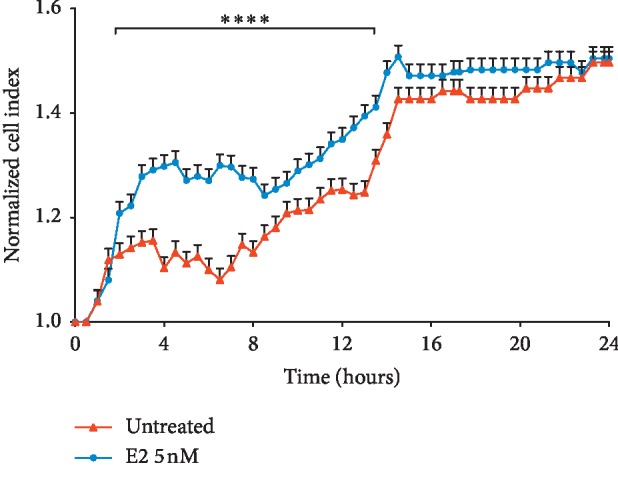
Effect of E2 in glioblastoma cell proliferation. E2 treatment resulted in a significant increase in cell proliferation in U87-MG cells following E2 5 nM treatment. A normalized cell index was performed for 24 hours by xCELLigence RTCA technology.

**Figure 2:**
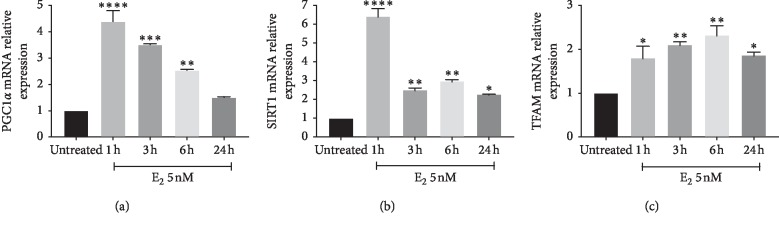
Effect of E2 on mitochondrial biogenesis. E2 resulted in a significant increase of PGC1α gene expression following 1 h treatment, and such an expression decreases in a time-dependent manner reaching the control levels following 24 h (Figure 2(a)). Consistently, SIRT1 and TFAM show a significant increase following 1 h of E2 treatment (Figures 2(b) and 2(c)). The calculated value of 2^‐ΔΔ^C^t^ in untreated controls is 1. Data are expressed as mean ± SD of at least four independent experiments. ^*∗*^*p* < 0.05; ^*∗∗*^*p* < 0.001; and ^*∗∗∗*^*p* < 0.0001.

**Figure 3:**
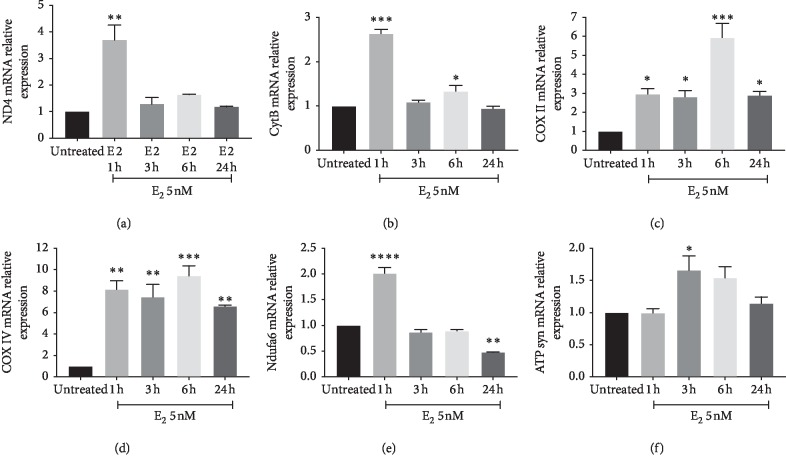
Effect of E2 on mitochondrial OXPHOS gene. E2 treatment significantly increased ND4, Cyb4, COXII, COXIV, COX, and NDUFA6 gene expression following 1 h treatment of E2 (Figures 3(a)–3(e)). Similarly, the treatment increases ATP synthase gene expression and peaks at 3 h (Figure 3(f)). The calculated value of 2^‐ΔΔ^C^t^ in untreated controls is 1. Data are expressed as mean ± SD of at least four independent experiments. ^*∗*^*p* < 0.05; ^*∗∗*^*p* < 0.001; and ^*∗∗∗*^*p* < 0.0001.

**Figure 4:**
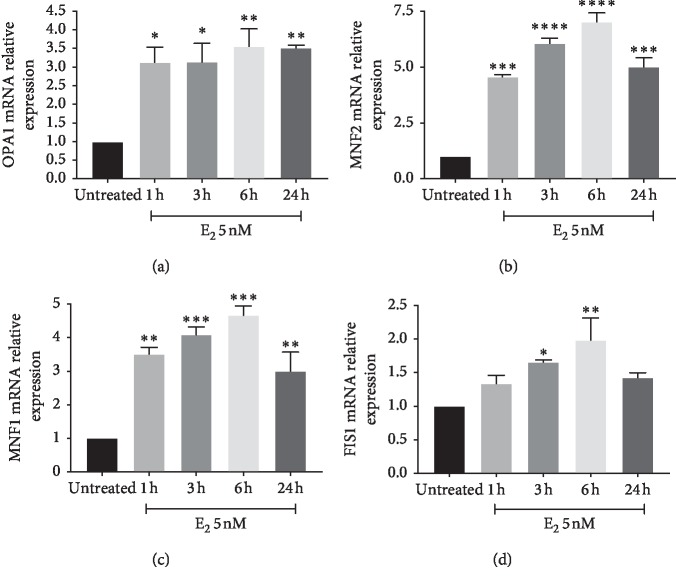
Effect of E2 on mitochondrial dynamics. E2 treatment increases OPA1, MNF2, and MNF1 gene expression following 1 hour of treatment (Figures 4(a)–4(c)). Consistently, E2 treatment increases FIS1 gene expression following 3 hours of treatment (Figure 4(d)). The calculated value of 2^‐ΔΔ^C^t^ in untreated controls is 1. Data are expressed as mean ± SD of at least four independent experiments. ^*∗*^*p* < 0.05; ^*∗∗*^*p* < 0.001; and ^*∗∗∗*^*p* < 0.0001.

**Figure 5:**
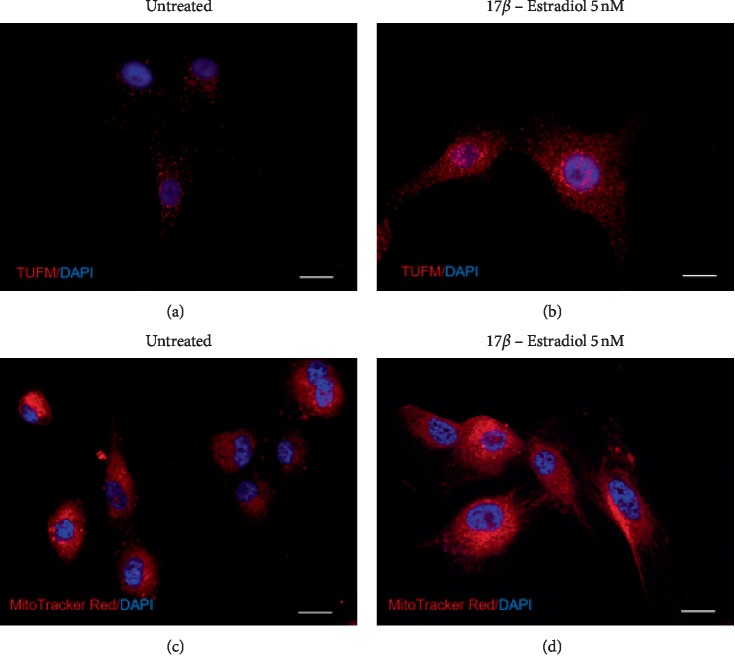
E2 increases mitochondrial mass in glioblastoma cells. The immunocytochemistry analysis demonstrates an increase in TFAM protein expression and mitochondrial network. Immunofluorescence staining of TFUM (red) was performed in U87-MG human glioblastoma cells in basal condition (Figure 5(a)) and after 24 hours of treatment with E2 (Figure 5(b)). The Mitotracker Red staining was performed in U87-MG human glioblastoma cells at basal condition (Figure 5(c)) and after 24 hours of treatment with E2 (Figure 5(d)). DAPI was used to stain the cell nucleus, and the scale bar is set as 10 *μ*m.

We firstly aimed at studying the effect of E2 on cell proliferation. As shown in Figure 1, E2 treatment resulted in a significant increase in cell proliferation in U87-MG cells, as showed by cell index performed by xCELLigence technology. Increased cell index was already significant following 3 hours (*p* < 0.001) of treatment with E2, and such effect was still evident following 9 hours of treatment (*p* < 0.001). We, therefore, investigated the effect of E2 on mitochondrial metabolism concerning mitochondrial biogenesis, oxidative phosphorylation, and dynamics. As shown in Figure 2(a), E2 resulted in a significant increase of PGC1*α* gene expression following 1 hour of treatment (*p* < 0.001), and such an expression decreases in a time-dependent manner reaching the control levels following 24 h. Consistently, we observed a significant increase in two additional biomarkers of mitochondrial biogenesis (i.e., SIRT1 and TFAM) (Figures 2(b) and 2(c)). This set of experiments showed that E2 resulted in a significant (*p* < 0.001) increase in SIRT1 and TFAM gene expression following 1 hour of E2 treatment, and such an increased expression was sustained during all other times of observation. As shown in Figure 3, E2 treatment also resulted in a significant change in the expression of genes involved in oxidative phosphorylation. E2 treatment significantly increased ND4, Cyb4, COXII, COXIV, COX, and NDUFA6 gene expression following 1-hour treatment of E2 (Figures 3(a)–3(e)). Similarly, ATP synthase gene expression significantly increased treatment and peaked 3 h following E2 pharmacological treatment (Figure 3(f)). Besides, E2 exhibited a significant effect on the expression controlling mitochondrial dynamics. E2 treatment resulted in a significant (*p* < 0.001) increase of OPA1, MNF2, and MNF1 gene expression following 1 hour of treatment (Figures 4(a)–4(c)). Consistently, E2 treatment resulted in a significant (*p* < 0.010) increase in FIS1 gene expression following 3 hours of E3 treatment (Figure 4(d)). Finally, these results were further confirmed by immunocytochemistry analysis, demonstrating increase TFAM protein expression and increased mitochondrial network as measured by mitotracker staining (Figures 5(a)–5(d)).

### 3.2. E2 Induces Nrf2 Nuclear Translocation and Increases Heme Oxygenase-1 Expression

**Figure 6:**
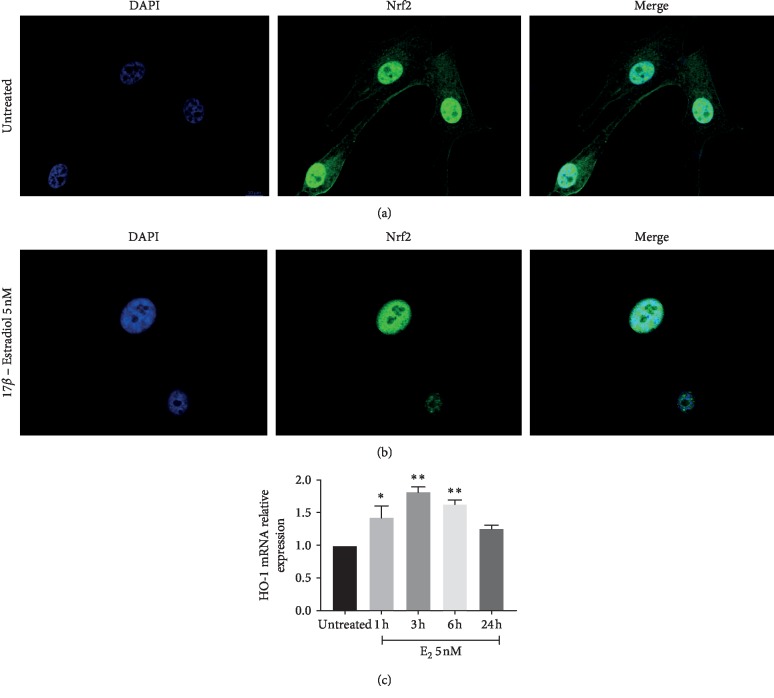
E2 increases HO-1 expression and induces Nrf2 nuclear translocation. E2 treatment increases Nrf2 nuclear translocation following 24 h treatment when compared with untreated cells (Figures 6(a) and 6(b)). Consistently, HO-1, one of the Nrf2-targeted genes, is upregulated following E2 treatment (*p* < 0.001) (Figure 6(c)). Immunofluorescence staining of Nrf2 (green) was performed in U87-MG human glioblastoma cells at basal condition (Figure 6(a)) and after 24 hours of treatment with E2 (Figure 6(b)). DAPI was used to stain the cell nucleus, and the scale bar is set as 10 *μ*m. Gene expression analysis of HO-1 was performed after 24 hours of treatment with E2 (5 nM) in glioblastoma cells. The calculated value of 2^‐ΔΔ^C^t^ in untreated controls is 1. Data are expressed as mean ± SD of at least four independent experiments (Figure 6(c)). ^*∗*^*p* < 0.05; ^*∗∗*^*p* < 0.001.

To assess the effect of E2 on the activation of pathways involved in chemoresistance mechanisms, we evaluated the nuclear translocation of Nrf2. Our data showed that E2 treatment resulted in a significant increase in nuclear translocation following 24 h treatment when compared to untreated cells (Figures 6(a) and 6(b)). Consistently with this observation, we also showed that HO-1, an Nrf2-targeted gene, was upregulated following E2 treatment (*p* < 0.001) (Figure 6(a)).

## 4. Discussion

Previous studies showed that high concentrations of estradiol, under low growth-stimulated conditions, inhibit cell proliferation and increase apoptosis in ER-positive breast cancer cells through the sustained activation of the JNK pathway [12, 22]. These findings emphasize the basis for the antitumor effects of high-dose estrogen therapy in postmenopausal women approximately 40 years ago [20]. Recently, high concentrations of estradiol were shown to trigger apoptosis in adrenal carcinoma cells [23], indicating that the mechanisms of these cytotoxic effects of estradiol remain to be further elucidated. Glioblastomas are the most aggressive type of brain tumors, with a poor prognosis and a limited response to chemotherapy and other therapeutic strategies [24, 25]. Failure of therapy arises from the resistance of tumor cells to therapy-induced apoptosis [26]; therefore, new drugs targeting alternative pathways are required. In the present study, E2 induces cell proliferation and the expression of genes involved in mitochondrial metabolism in glioblastoma cells. Estradiol, the predominant form of estrogen, mediates its effects via the activation of intracellular signaling pathways on neurons and glial cells [27]. Previous studies concerning the effects of estrogens in cancer cells exhibited controversial results [28]. With regard to glioblastoma, epidemiological evidence suggests an E2 tumor suppressor role [29]. The rate of the development of glioblastoma is increased in men: women aged 15–49 years (women of reproductive age) have a survival advantage compared with men and postmenopausal women [29–31]. These results suggest that estrogens are involvement in the suppression of glioblastoma, but how they could do it is poorly understood. By contrast, our data suggest that E2 induces cell proliferation in the U87-MG glioblastoma cell line. However, a different expression of ER*β* may explain, at least in part, the discrepancy with previously published reports. In this regard, multiple ER*β* isoforms exist and may have distinct roles in various cancers [32–34]. The ER*β*2 isoform is increased in chronic lymphatic leukemia, prostate cancer, non-small-cell lung cancer, breast cancer, and ovarian cancer [35]. The worsening disease-free survival and overall survival of patients were correlated with ER*β*2 expression in patients treated with tamoxifen [36]. Moreover, ER*β*2 is involved also in the metastasis of prostate cancer [37]. In addition, ER*β*3 has restricted to testis [38]. ER*β*5 is overexpressed in ovarian cancer and prostate cancer and associated with poor prognosis [39], while ER*β*5 expression is associated with good prognosis in non-small-cell lung cancer and confers sensitivity to chemotherapeutic agent-induced apoptosis in breast cancer cells [39]. Several authors advanced that ER*β*5 was highly expressed in primary and established GBM cells compared to ER*β*1 and ER*β*2, with ER*β*4 [34, 40]. The data regarding the effect of E2 on glioblastoma progression are further supported by our results showing that E2 induces Nrf2 nuclear translocation and HO-1 expression. Estradiol also exerts nongenomic rapid actions via direct interaction of estradiol with plasma-associated ERs and the activation of second messenger pathways [41]. The late and sustained effects of estradiol described in this study suggest that nongenomic rapid actions of estradiol are not involved. In this regard, it has become evident that malignant cells benefit from having increased Nrf2 pathway activity: this was first observed in lung cancer [42], as well as subsequently in many other cancer types, such as pancreatic, ovarian, liver, and gallbladder cancers [43]. Aberrant Keap1-Nrf2 signaling leads to radio- and chemoresistance and provides a growth advantage to cancer cells, due to the constitutive expression of cytoprotective genes [44]. Multiple mechanisms for Nrf2 overactivation have been found, such as somatic mutations in either KEAP1 or NFE2L2, deletion of exon 2 of NFE2L2, aberrant expression of inhibitory proteins, and transcriptional induction by oncogenes and hormones [45]. Previous results demonstrated that, in GBM cells, inhibition of Nrf2 and p62 decreased tumorigenic properties, such as cell invasion and anchorage-independent growth [46]. Furthermore, Nrf2 could also function as a key balancing factor in metabolic reprogramming, as Nrf2 can regulate both energy metabolism and antioxidant response to ROS to favor glioma growth and development. Our results are consistent with these observations and showed that E2 resulted in a significant increase in the expression of genes involved in mitochondrial metabolism, biogenesis, and dynamics. Furthermore, our results showed that E2 resulted in a significant increase of HO-1, which is associated with increased chemoresistance and proliferative phenotype [47, 48], thus further confirming our observations. Our data showed that E2 plays an important role in GBM progression, improving the mitochondrial fitness, highlighting its role in resistant mechanisms to the therapies: this can lead to a new therapeutic strategy for future studies.

## Figures and Tables

**Table 1:** List of qRT-PCR primers.

Gene of interest	Forward primer (5′ ⟶ 3′)	Reverse primer (5′ ⟶ 3′)
PGC1α	ATGAAGGGTACTTTTCTGCCCC	GGTCTTCACCAACCAGAGCA
SIRT1	AGGCCACGGATAGGTCCATA	GTGGAGGTATTGTTTCCGGC
TFAM	CCGAGGTGGTTTTCATCTGT	AGTCTTCAGCTTTTCCTGCG
ND4	CCAGTGGAATGCCTTGCCTA	TTGATCGCGGTGAGATTCCC
CyB	ACGAGCCACCGAAACAGAAT	ACGATTTTCGCCAGTCACCT
COX II	ACGACCTCGATGTTGGATCA	ATCATTTACGGGGGAAGGCG
COX IV	GCGGCAGAATGTTGGCTAC	AGACAGGTGCTTGACATGGG
NDUFA6	CAGTCGGGACATGAACGAGG	GAATTGGTGCACAGTGTTCG
ATP synthase	CCGCCTTCCGCGGTATAATC	ATGTACGCGGGCAATACCAT
OPA1	AGGAGCTCATCTGTTTGGAGTC	GCTCACCAAGCAGACCCTTT
MNF2	GCGGAGACTCATAATGGCAGA	TCCGAGATAGCACCTCACCA
MNF1	ATGCAGTGGGAGTCCGAGC	CAGGGACATTGCGCTTCAC
FIS1	AAGAAAGATGGACTCGTGGGC	CCGCGTCTCCTTCAGGATTT
HO-1	AAGACTGCGTTCCTGCTCAA	GGGCAGAATCTTGCACTTTGT
*β*-Actin	CCTTTGCCGATCCGCCG	AACATGATCTGGGTCATCTTCTCGC

## Data Availability

The data used to support the findings of this study are available from the corresponding author upon request.

## References

[1] Lefranc F., Le Rhun E., Kiss R., Weller M. (2018). Glioblastoma quo vadis: will migration and invasiveness reemerge as therapeutic targets?. *Cancer Treatment Reviews*.

[2] Ventura E., Weller M., Burghardt I. (2017). Cutting edge: ERK1 mediates the autocrine positive feedback loop of TGF-*β* and furin in glioma-initiating cells. *The Journal of Immunology*.

[3] Haas-Kogan D. A., Prados M. D., Tihan T. (2005). Epidermal growth factor receptor, protein kinase B/Akt, and glioma response to erlotinib. *JNCI: Journal of the National Cancer Institute*.

[4] Lin L., Cai J., Jiang C. (2017). Recent advances in targeted therapy for glioma. *Curr Med Chem*.

[5] Maugeri G., D’amico A. G., Magro G. (2015). Expression profile of parkin isoforms in human gliomas. *International Journal of Oncology*.

[6] DeBerardinis R. J., Chandel N. S. (2016). Fundamentals of cancer metabolism. *Science Advances*.

[7] Yague J. G., Lavaque E., Carretero J., Azcoitia I., Garcia-Segura L. M. (2004). Aromatase, the enzyme responsible for estrogen biosynthesis, is expressed by human and rat glioblastomas. *Neuroscience Letters*.

[8] Sribnick E. A., Ray S. K., Banik N. L. (2006). Estrogen prevents glutamate-induced apoptosis in C6 glioma cells by a receptor-mediated mechanism. *Neuroscience*.

[9] Hui A.-M., Zhang W., Chen W. (2004). Agents with selective estrogen receptor (ER) modulator activity induce ApoptosisIn vitroandIn vivoin ER-negative glioma cells. *Cancer Research*.

[10] Moodbidri M. S., Shirsat N. V. (2005). Activated JNK brings about accelerated apoptosis of Bcl-2-overexpressing C6 glioma cells on treatment with tamoxifen. *Journal of Neurochemistry*.

[11] Tian F., Wu H., Li Z. (2009). Activated PKCα/ERK1/2 signaling inhibits tamoxifen-induced apoptosis in C6 cells. *Cancer Investigation*.

[12] Altiok N., Koyuturk M., Altiok S. (2007). JNK pathway regulates estradiol-induced apoptosis in hormone-dependent human breast cancer cells. *Breast Cancer Research and Treatment*.

[13] Torres M. J., Kew K. A., Ryan T. E. (2018). 17*β*-Estradiol directly lowers mitochondrial membrane microviscosity and improves bioenergetic function in skeletal muscle. *Cell Metabolism*.

[14] Galmés-Pascual B. M., Nadal-Casellas A., Bauza-Thorbrügge M. (2017). 17*β*-estradiol improves hepatic mitochondrial biogenesis and function through PGC1B. *Journal of Endocrinology*.

[15] Mattingly K. A., Ivanova M. M., Riggs K. A., Wickramasinghe N. S., Barch M. J., Klinge C. M. (2008). Estradiol stimulates transcription of nuclear respiratory factor-1 and increases mitochondrial biogenesis. *Molecular Endocrinology*.

[16] Chou C.-H., Chen S.-U., Chen C.-D. (2020). Mitochondrial dysfunction induced by high estradiol concentrations in endometrial epithelial cells. *The Journal of Clinical Endocrinology & Metabolism*.

[17] Hirano S., Furutama D., Hanafusa T. (2007). Physiologically high concentrations of 17*β*-estradiol enhance NF-*κ*B activity in human T cells. *American Journal of Physiology-Regulatory, Integrative and Comparative Physiology*.

[18] McCarthy M. M. (2009). The two faces of estradiol: effects on the developing brain. *The Neuroscientist*.

[19] Waters E. M., Simerly R. B. (2009). Estrogen induces caspase-dependent cell death during hypothalamic development. *Journal of Neuroscience*.

[20] Lewis-Wambi J. S., Jordan V. C. (2009). Estrogen regulation of apoptosis: how can one hormone stimulate and inhibit?. *Breast Cancer Research*.

[21] Sacerdoti D., Colombrita C., Ghattas M. H. (2005). Heme oxygenase-1 transduction in endothelial cells causes downregulation of monocyte chemoattractant protein-1 and of genes involved in inflammation and growth. *Cell Mol Biol (Noisy-Le-Grand)*.

[22] Altiok N., Ersoz M., Koyuturk M. (2011). Estradiol induces JNK-dependent apoptosis in glioblastoma cells. *Oncology Letters*.

[23] Prieto L., Brown J., Perez-Stable C., Fishman L. (2008). High dose 17 *β*-estradiol and the α-estrogen agonist PPT trigger apoptosis in human adrenal carcinoma cells but the *β*-estrogen agonist DPN does not. *Hormone and Metabolic Research*.

[24] Minniti G., Muni R, Lanzetta G, Marchetti P, Enrici R. M (2009). Chemotherapy for glioblastoma: current treatment and future perspectives for cytotoxic and targeted agents. *Anticancer Research*.

[25] Quick A., Patel D., Hadziahmetovic M., Chakravarti A., Mehta M. (2010). Current therapeutic paradigms in glioblastoma. *Reviews on Recent Clinical Trials*.

[26] Lino M., Merlo A. (2009). Translating biology into clinic: the case of glioblastoma. *Current Opinion in Cell Biology*.

[27] Pozzi S., Benedusi V., Maggi A., Vegeto E. (2006). Estrogen action in neuroprotection and brain inflammation. *Annals of the New York Academy of Sciences*.

[28] Russo M., Russo G. L., Daglia M. (2016). Understanding genistein in cancer: the “good” and the “bad” effects: a review. *Food Chemistry*.

[29] Parés D. (2010). Adequate management of postoperative pain in surgery for hemorrhoidal disease. *Cirugía Española (English Edition)*.

[30] Kabat G. C., Park Y., Hollenbeck A. R., Schatzkin A., Rohan T. E. (2011). Reproductive factors and exogenous hormone use and risk of adult glioma in women in the NIH-AARP Diet and Health Study. *International Journal of Cancer*.

[31] Anic G. M., Madden M. H., Nabors L. B. (2014). Reproductive factors and risk of primary brain tumors in women. *Journal of Neuro-Oncology*.

[32] Leung Y.-K., Mak P., Hassan S., Ho S.-M. (2006). Estrogen receptor (ER)-beta isoforms: a key to understanding ER-beta signaling. *Proceedings of the National Academy of Sciences*.

[33] Thomas C., Gustafsson J.-Å. (2011). The different roles of ER subtypes in cancer biology and therapy. *Nature Reviews Cancer*.

[34] Liu J., Sareddy G. R., Zhou M. (2018). Differential effects of estrogen receptor beta isoforms on glioblastoma progression. *Cancer Research*.

[35] Dey P., Barros R. P. A., Warner M., Ström A., Gustafsson J.-Å. (2013). Insight into the mechanisms of action of estrogen receptor *β* in the breast, prostate, colon, and CNS. *Journal of Molecular Endocrinology*.

[36] Baek J.-M., Chae B.-J., Song B.-J., Jung S.-S. (2015). The potential role of estrogen receptor *β*2 in breast cancer. *International Journal of Surgery*.

[37] Dey P., Jonsson P., Hartman J., Williams C., Ström A., Gustafsson J.-Å. (2012). Estrogen receptors *β*1 and *β*2 have opposing roles in regulating proliferation and bone metastasis genes in the prostate cancer cell line PC3. *Molecular Endocrinology*.

[38] Moore J. T., McKee D. D., Slentz-Kesler K. (1998). Cloning and characterization of human estrogen receptor *β* isoforms. *Biochemical and Biophysical Research Communications*.

[39] Leung Y.-K., Lam H.-M., Wu S. (2010). Estrogen receptor *β*2 and *β*5 are associated with poor prognosis in prostate cancer, and promote cancer cell migration and invasion. *Endocrine-Related Cancer*.

[40] Li W., Winters A., Poteet E. (2013). Involvement of estrogen receptor *β*5 in the progression of glioma. *Brain Research*.

[41] Levin E. R. (2009). Plasma membrane estrogen receptors. *Trends in Endocrinology & Metabolism*.

[42] Padmanabhan B., Tong K. I., Ohta T. (2006). Structural basis for defects of Keap1 activity provoked by its point mutations in lung cancer. *Molecular Cell*.

[43] Lister A., Nedjadi T., Kitteringham N. R. (2011). Nrf2 is overexpressed in pancreatic cancer: implications for cell proliferation and therapy. *Molecular Cancer*.

[44] Lau A., Wang X.-J., Zhao F. (2010). A noncanonical mechanism of Nrf2 activation by autophagy deficiency: direct interaction between Keap1 and p62. *Molecular and Cellular Biology*.

[45] Goldstein L. D., Lee J., Gnad F. (2016). Recurrent loss of NFE2L2 exon 2 is a mechanism for Nrf2 pathway activation in human cancers. *Cell Reports*.

[46] Pölönen P., Deen A. J., Leinonen H. M. (2019). Nrf2 and SQSTM1/p62 jointly contribute to mesenchymal transition and invasion in glioblastoma. *Oncogene*.

[47] Gandini N. A., Fermento M. E., Salomón D. G. (2014). Heme oxygenase-1 expression in human gliomas and its correlation with poor prognosis in patients with astrocytoma. *Tumor Biology*.

[48] Barbagallo I., Giallongo C., Volti G. L. (2019). Heme oxygenase inhibition sensitizes neuroblastoma cells to carfilzomib. *Molecular Neurobiology*.

